# Clinical and Radiological Outcomes After Curative-Intent Induction Chemotherapy for Primary Advanced Oral Cavity Squamous Cell Carcinoma

**DOI:** 10.3390/jcm15208165

**Published:** 2026-10-20

**Authors:** Paola Lovino Camerino, I-Ching Chen, Cheng-Lung Hsu, Chi-Kuang Young, Chih-Yung Yang, Yu-Feng Hu, Hung-Ming Wang, Valentina Campagnari, Chun-Ta Liao, Chung-Kan Tsao, Shiang-Fu Huang

**Affiliations:** 1 Department of Otolaryngology, Head and Neck Surgery, Chang Gung Memorial, Linkou 333423, Taiwan; lovino.pa@gmail.com (P.L.C.);; 2 Maxillofacial Surgery Unit, Interdisciplinary Department of Medicine, University of Bari “Aldo Moro”, 70124 Bari, Italy; 3 Department of Medical Education, Chang Gung Memorial Hospital, Taoyuan 333423, Taiwan; 4 Division of Hemato-Oncology, Department of Internal Medicine, Chang Gung Memorial, Linkou 333423, Taiwan; 5 School of Medicine, Medical College, Chang Gung University, Kwei-Shan, Taoyuan 333323, Taiwan; 6 Department of Otolaryngology, Head and Neck Surgery, Chang Gung Memorial, Keelung Branch, Keelung 204201, Taiwan; 7 Department of Otolaryngology, Hsin-Chu MacKay Memorial Hospital, Hsin-Chu 300044, Taiwan; 8 Department of Plastic and Reconstructive Surgery, Chang Gung Memorial Hospital, Taoyuan 333423, Taiwan; 9 Graduate Institute of Clinical Medical Sciences, Chang Gung University, Taoyuan 333002, Taiwan

**Keywords:** oral cavity squamous cell carcinoma, induction chemotherapy, nodal burden, unidimensional response, surgery, overall survival

## Abstract

**Background/Objectives:** The role of induction chemotherapy (IC) in advanced oral cavity squamous cell carcinoma (OCSCC) remains controversial. This study evaluated patient-level changes in primary-tumour and cervical nodal dimensions, subsequent definitive treatment, and survival after curative-intent IC. **Methods:** We retrospectively analysed 38 patients with primary, non-metastatic OCSCC treated with curative-intent IC between 2009 and 2024. Recurrent disease, second primary tumours, non-squamous histology, non-oral primary sites, baseline metastatic disease, direct chemoradiotherapy, and palliative systemic treatment were excluded. A prespecified simplified unidimensional radiological analysis was performed. The maximum axial primary-tumour diameter, the largest baseline cervical node, and total recorded nodal burden were compared before and after IC. Baseline nodes absent after IC were coded as 0 mm only when disappearance was confirmed on post-IC anatomical imaging. The overall survival (OS) was measured from IC initiation. **Results:** Primary-tumour measurements were evaluable in 25 patients. Median diameter decreased from 55.0 to 46.0 mm, corresponding to a median change of −13.5% (*p* = 0.049). The largest baseline node was evaluable in 19 patients and decreased from 17.0 to 13.0 mm, a median change of −16.7% (*p* = 0.004). Total nodal burden changed by a median of −8.3% (*p* = 0.257). Primary-tumour change did not differ significantly from largest-node change (*p* = 0.073) or total nodal-burden change (*p* = 0.860). TP and TPF showed no difference in primary-tumour change (*p* = 0.954). During a median follow-up of 40.4 months, 16 patients died. Median OS was 45.1 months and 5-year OS was 35.6%. **Conclusions:** IC produced heterogeneous, generally modest radiological changes. The largest baseline node decreased significantly, whereas total nodal burden did not. These data do not establish the superiority of one regimen or a causal survival benefit from surgery and should be considered hypothesis-generating.

## 1. Introduction

Locally advanced oral cavity squamous cell carcinoma is primarily managed with surgical resection followed by risk-adapted radiotherapy or chemoradiotherapy. Primary non-surgical treatment generally remains reserved for patients in whom surgery is not feasible or is declined. Despite advances in surgical reconstruction, radiotherapy planning, and systemic treatment, outcomes remain unsatisfactory in patients with extensive T4 or bulky nodal disease [1,2].

Currently, several trials are investigating the effect of neoadjuvant chemotherapy in operable non-small-cell lung cancer. Moreover, it is already an established clinical practice to downsize tumours, facilitate organ-preserving surgery, and improve the overall survival (OS) in breast cancer, cervical cancer, and osteosarcoma [3,4,5,6].

Despite this evidence for non-head and neck malignancies, the role of induction chemotherapy (IC) in OCC is still debated with many unanswered questions.

Induction chemotherapy has been investigated as a strategy for tumour cytoreduction, conversion to resectability, reduction in surgical extent, and biological selection. However, randomised studies specifically conducted in resectable OCSCC did not demonstrate a consistent overall-survival advantage over upfront surgery [7,8,9,10,11]. Retrospective data suggest that IC may nevertheless have a role in selected borderline-resectable cases or as a bridge to definitive treatment [11,12,13,14,15].

TPF remains the best-established induction regimen in locoregionally advanced head and neck squamous cell carcinoma, but its toxicity and treatment burden have motivated the use of taxane–platinum combinations in selected patients [16,17,18,19,20,21,22,23]. Direct retrospective comparisons are difficult because regimen choice is affected by comorbidity, physician preference, institutional protocols, and the treatment era.

The systemic-treatment landscape has changed substantially with immune-checkpoint inhibition. A phase 2 trial of neoadjuvant nivolumab with or without ipilimumab showed feasibility and pathological activity in untreated OCSCC [24]. More recently, KEYNOTE-689 demonstrated improved event-free survival with perioperative pembrolizumab added to surgery and adjuvant therapy in locally advanced HNSCC, leading to regulatory approval for PD-L1-positive resectable disease [25,26]. Conventional IC data must therefore be interpreted as historical and hypothesis-generating rather than as evidence for a universal contemporary standard.

The objectives of this study were to quantify patient-level unidimensional changes in the primary tumour, largest baseline cervical node, and total nodal burden; compare primary and nodal changes; describe definitive treatment after IC; and estimate overall survival without making causal treatment claims.

## 2. Materials and Methods

### 2.1. Study Design and Eligibility

This retrospective single-centre cohort study was conducted at the Department of Otolaryngology—Head and Neck Surgery, Linkou Chang Gung Memorial Hospital. The study was approved by the institutional review board (No. 202500899B0) and conducted in accordance with the Declaration of Helsinki. Medical records of patients treated between 2009 and 2024 were reviewed. The objectives were to quantify patient-level unidimensional changes in the primary tumour, largest baseline cervical node, and total nodal burden; compare primary and nodal changes; to describe definitive treatment after IC; and estimate overall survival.

Eligible patients had histologically confirmed primary OCSCC, no distant metastatic disease at IC initiation, and received IC with curative intent. Exclusion criteria were recurrent oral cavity disease, second primary tumours, non-squamous histology, non-oral cavity tumours, metastatic disease at baseline, direct definitive chemoradiotherapy without IC, or palliative systemic treatment at baseline. Patients who progressed or received palliative treatment after curative-intent IC remained in the cohort.

### 2.2. Induction Treatment and Subsequent Management

IC regimens included TPF, TP, and PUL–bleomycin, administered according to institutional practice, patient characteristics, comorbidity, and treatment era. TPF consisted of docetaxel, cisplatin, and 5-fluorouracil; TP consisted of docetaxel and cisplatin; and PUL–bleomycin consisted of cisplatin, oral tegafur, leucovorin, and bleomycin [16,27,28,29]. The number of cycles and dose modifications were determined clinically.

After IC, patients were reassessed by the multidisciplinary team. Surgery was performed when the disease was technically operable, the patient was clinically fit, and surgery was accepted. Patients not undergoing surgery received definitive radiotherapy or chemoradiotherapy according to clinical status. Surgery was analysed as a post-baseline treatment pathway and not as a randomised exposure.

### 2.3. Radiological Measurements and Nodal Disappearance

Pre- and post-IC CT or MRI studies were reviewed using unidimensional measurements. Full RECIST 1.1 criteria were not claimed because complete target-lesion sums and non-target lesion assessments could not be reconstructed consistently [30]. Unidimensional measurement was selected because maximum-diameter assessment had shown high agreement with bidimensional response classification and may adequately characterise change in solid tumours [31].

The maximum axial diameter of the primary tumour was recorded. Up to three ipsilateral and three contralateral pathological cervical nodes were recorded using short-axis diameter. Three patient-level nodal endpoints were derived: the largest baseline node; the sum of the short-axis diameters of all recorded nodes; and the individual percentage change in each endpoint.

A baseline-measured node that was no longer visible or measurable on a valid post-IC anatomical study after source-data review was classified as radiological nodal disappearance and assigned a post-IC diameter of 0 mm for the disappearance-aware analysis. A blank post-treatment value was not assigned 0 when anatomical follow-up imaging was unavailable or non-evaluable. Baseline N0 patients were classified as not applicable rather than missing for dimensional nodal analysis. Newly recorded post-IC nodes were included in post-IC total nodal burden and documented as newly appearing nodes. This approach was explicitly descriptive and was not labelled RECIST response.

### 2.4. Outcomes

The principal radiological outcomes were absolute and percentage changes in primary-tumour diameter, largest baseline-node diameter, and total nodal burden. Secondary outcomes included subsequent surgery and OS. OS was defined from IC initiation to death from any cause or last follow-up.

### 2.5. Statistical Analysis

Continuous variables were summarised using medians and ranges or interquartile ranges, and categorical variables using counts and percentages. Paired pre/post changes were tested with the Wilcoxon signed-rank test. Primary-tumour percentage change was compared with largest-node and total-nodal-burden percentage change using paired Wilcoxon tests. Associations between continuous response measures were explored using Spearman correlation. TP and TPF were compared using the Mann–Whitney U test. PUL–bleomycin was not included in formal regimen comparisons because of its small evaluable sample. The OS was estimated by Kaplan–Meier methods and median follow-up by reverse Kaplan–Meier. To explore whether radiological shrinkage was associated with OS while limiting guarantee-time bias, univariable landmark Cox models were calculated from the post-IC imaging date among patients with an evaluable measurement; hazard ratios were expressed per 10% greater reduction. Surgery was not entered as a conventional fixed baseline covariate because it occurred after IC and was affected by response, resectability, fitness, and survival to reassessment. Analyses were exploratory, two-sided, and no multiplicity adjustment was applied. Given the cohort size and number of deaths, no multivariable survival model was prespecified.

## 3. Results

### 3.1. Cohort Characteristics

The final cohort included 38 patients. Median age was 56.5 years and 35 (92.1%) were male. TP was administered to 20 patients, TPF to 14, and PUL–bleomycin to 4. Twenty-four patients underwent surgery after IC (Table 1).

### 3.2. Primary-Tumour Response

**Figure 1:**
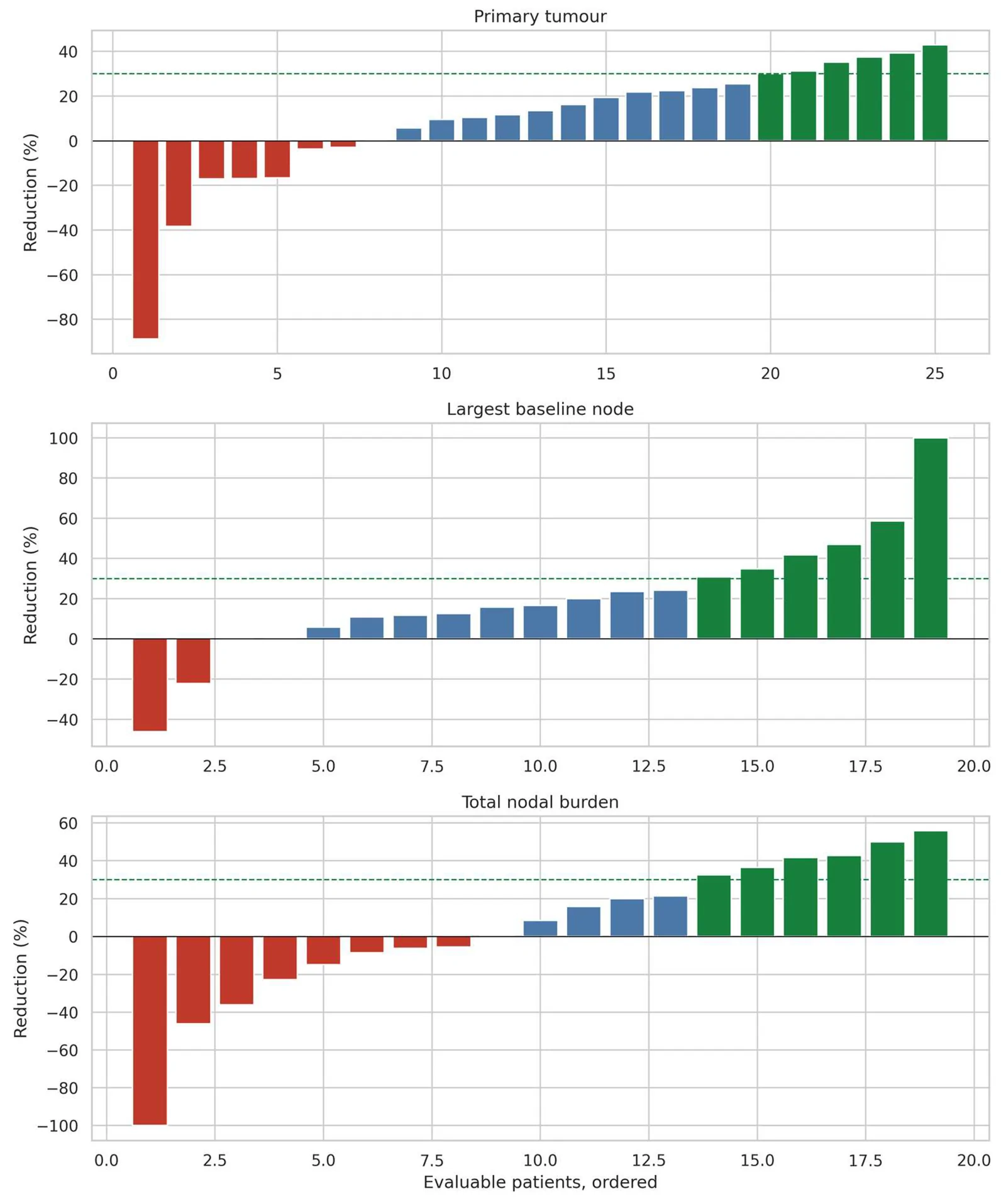
Individual unidimensional percentage reductions in the primary tumour, largest baseline node, and total nodal burden. Positive values indicate shrinkage. The 30% line is descriptive and is not presented as a RECIST threshold.

Primary-tumour measurements were evaluable in 25 patients. Median maximum diameter decreased from 55.0 to 46.0 mm. Median percentage change was −13.5% (Wilcoxon *p* = 0.049). Individual changes were heterogeneous, including both substantial shrinkage and progression (Figure 1).

### 3.3. Largest-Node and Total Nodal-Burden Response

The largest baseline node was evaluable in 19 patients. The median short-axis diameter decreased from 17.0 to 13.0 mm, with a median percentage change of −16.7% (*p* = 0.004). Six baseline nodes in six patients were coded as radiological disappearance and assigned 0 mm in the disappearance-aware analysis.

The total nodal burden was evaluable in 19 patients. The median-summed short-axis diameter changed from 28.0 to 30.0 mm. The median percentage change was −8.3% and was not statistically significant (*p* = 0.257). Eleven post-IC nodal measurements without a corresponding baseline measurement were included as newly recorded nodes in post-IC total burden. The difference between the reduction in the largest node and the absence of a significant reduction in total burden indicates that assessment of a single dominant node may not capture the entire nodal disease pattern.

**Figure 2:**
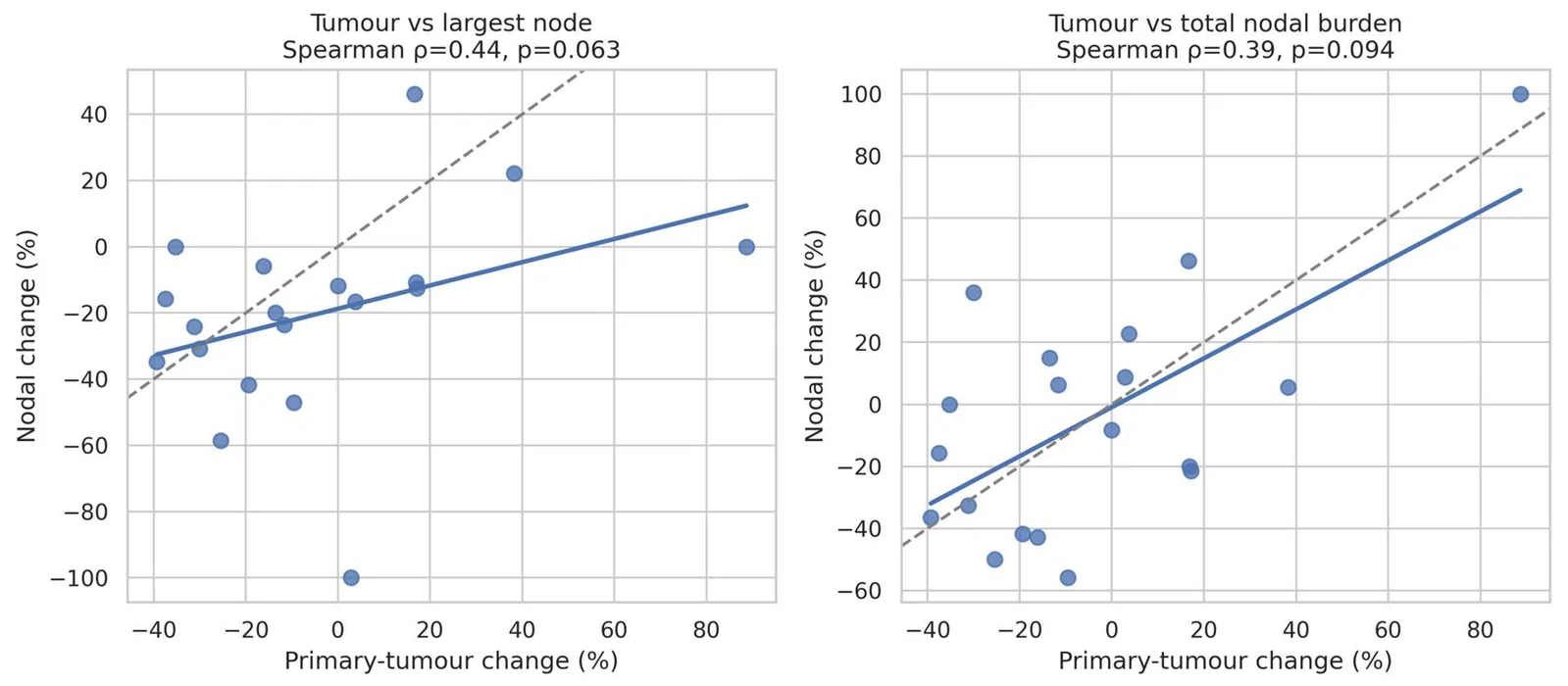
Patient-level association between primary-tumour change and largest-node or total-nodal-burden change. Negative values indicate shrinkage. The solid lines depict the regression lines between primary tumor change and nodal change.

Among 19 patients with paired primary-tumour and largest-node measurements, the changes did not differ significantly (*p* = 0.073); the Spearman correlation was ρ = 0.435 (*p* = 0.063). Among 19 patients evaluable for primary tumour and total nodal burden, the percentage changes also did not differ (*p* = 0.860), with ρ = 0.395 (*p* = 0.094) (Figure 2, Table 2 and Table 3).

### 3.4. Exploratory Regimen Comparison

Thirteen TP-treated and 11 TPF-treated patients were evaluable for primary-tumour change. There was no evidence of a difference between regimens (Mann–Whitney *p* = 0.954). Given the limited sample, non-random treatment allocation, treatment-era effects, and the absence of complete toxicity data, no conclusion regarding comparative efficacy or tolerability was made.

### 3.5. Survival

**Figure 3:**
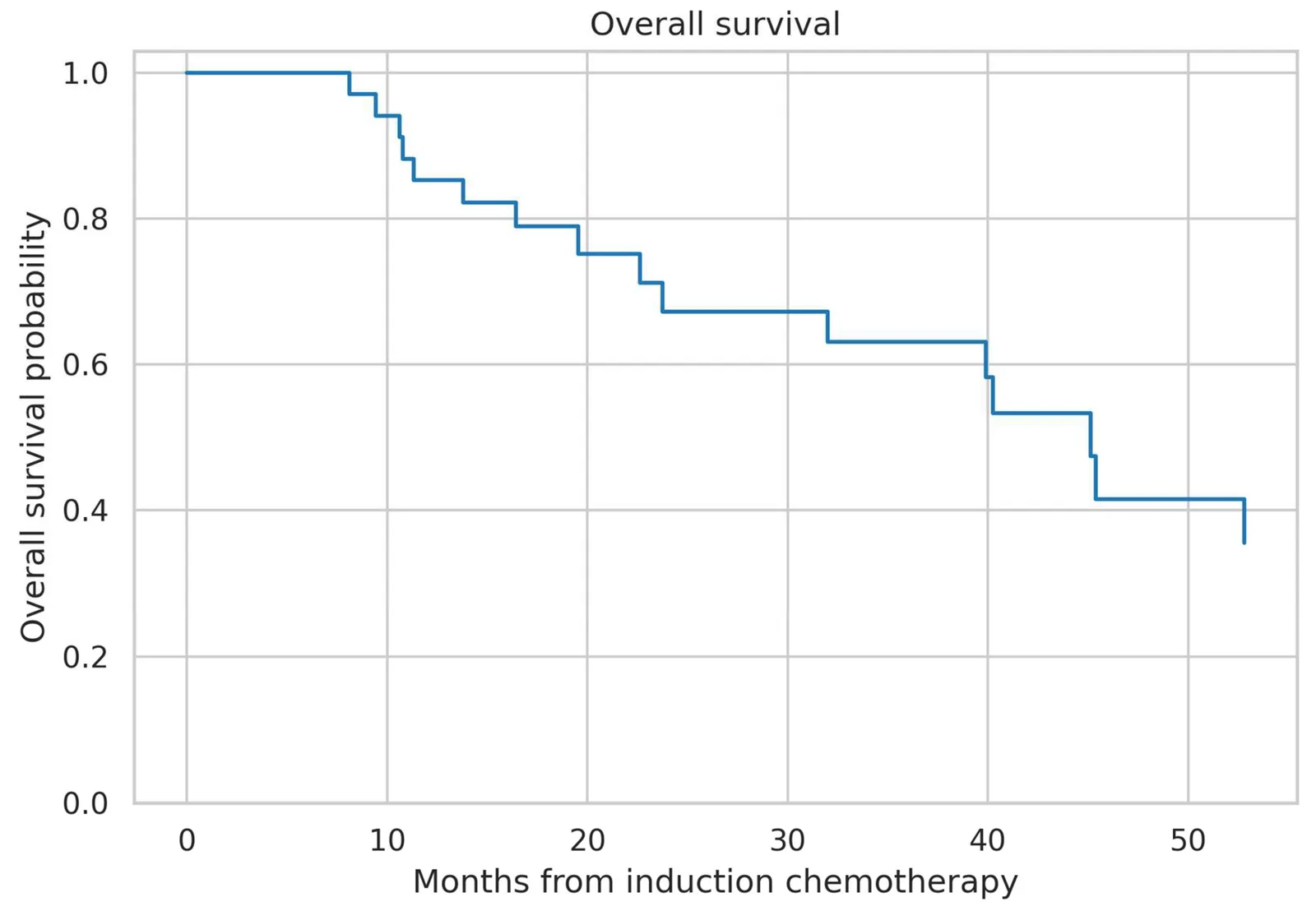
Overall survival from initiation of induction chemotherapy.

During a median follow-up of 40.4 months, 16 patients died, including 13 disease-related deaths and 3 deaths from other causes. The median OS from IC initiation was 45.1 months. The estimated OS was 85.3% at 1 year, 63.1% at 3 years, and 35.6% at 5 years (Figure 3).

In exploratory landmark Cox analyses beginning at the post-IC imaging assessment, radiological reduction was not associated with OS. The hazard ratio per 10% greater reduction was 1.03 for the primary tumour (95% CI, 0.83–1.29; *p* = 0.780; *n* = 25; 11 deaths), 1.21 for the largest baseline node (95% CI, 0.94–1.57; *p* = 0.141; *n* = 19; 9 deaths), and 1.13 for the total nodal burden (95% CI, 0.89–1.42; *p* = 0.320; *n* = 19; 9 deaths). These estimates were imprecise and considered hypothesis-generating.

Survival according to surgery was not interpreted causally because surgery was determined after IC and was affected by resectability, response, fitness, patient preference, and survival to reassessment (Table 4).

## 4. Discussion

This study provides a patient-level assessment of radiological change after curative-intent IC in a homogeneous cohort of primary, non-metastatic OCSCC. Three findings are clinically relevant. First, primary-tumour shrinkage was heterogeneous and modest at the cohort level. Second, nodal response depended on the metric selected: the largest baseline node decreased significantly, whereas the total nodal burden did not. Third, neither the tumour nor nodal shrinkage was associated with OS in exploratory landmark analyses. Together, these findings suggest that dimensional response after IC captures only one aspect of tumour behaviour and should not be considered a validated surrogate for long-term outcome.

The magnitude and heterogeneity of the primary-tumour change are consistent with the uncertain role of IC in OCSCC. The randomised trials by Licitra et al. and Zhong et al. did not demonstrate an OS advantage from adding preoperative chemotherapy to surgery-based treatment, and long-term follow-up and meta-analysis confirmed the absence of a consistent population-level survival benefit [7,8,9,11]. Nevertheless, these studies suggested potential secondary benefits, including reduced mandibular resection or postoperative radiotherapy in selected patients, and better prognoses among patients achieving pathological complete response [8,9]. More recently, Fu et al. reported objective responses in only a minority of 167 patients receiving neoadjuvant TPF, with most patients having stable or progressive disease [32]. Our median primary-tumour reduction of 13.5%, combined with the broad patient-level distribution, similarly argues against describing IC as uniformly cytoreductive in oral cavity disease.

The nodal findings add a distinct perspective. The significant reduction in the largest baseline node could suggest activity against dominant nodal disease; however, this interpretation changed when the entire recorded nodal burden was considered. The total burden did not decrease significantly, partly because the shrinkage or disappearance of one node could coexist with the persistence or emergence of other nodes. Fu et al. likewise observed that bulky or necrotic nodal disease frequently showed limited response or progression after TPF [32]. Biological heterogeneity between nodal deposits, variation in perfusion and hypoxia, and the coexistence of treatment-sensitive and resistant subclones provide plausible explanations for discordant responses among nodes and between primary and nodal sites [33]. These observations support reporting both dominant-node and multi-node burden rather than relying on a single representative node.

Although unidimensional measurements have demonstrated strong agreement with bidimensional response assessment [31], our approach is not equivalent to RECIST 1.1 because full target-lesion sums, non-target lesions, and standardised assessment of new lesions were unavailable [30]. The results should therefore be interpreted as quantitative exploratory imaging endpoints.

No difference in primary-tumour change was detected between TP and TPF. TPF is the most extensively studied induction regimen in locoregionally advanced HNSCC and demonstrated superiority to PF in landmark trials, although those studies included heterogeneous head and neck subsites and were not designed specifically for oral cavity cancer [16,17,18,23]. Our comparison involved only 13 TP-treated and 11 TPF-treated evaluable patients, was non-randomised, and was confounded by treatment era and clinical selection. Furthermore, toxicity was not reconstructed systematically. The present data therefore neither establish equivalence nor support substituting TP for TPF; they indicate only that no radiological difference was detectable in this small cohort.

Radiological shrinkage was not associated with OS in the landmark analyses. However, the absence of association should be interpreted cautiously because only 11 deaths occurred among patients evaluable for the primary-tumour model and nine in each nodal model. Pathological complete response has shown a prognostic value in long-term randomised data [9], but radiological size reduction is not interchangeable with pathological response and may fail to capture viable microscopic disease, extranodal extension, treatment-resistant subclones, or distant progression.

The treatment pathway after IC is also highly selected. Patients underwent surgery only if they survived induction, remained or became technically operable, were medically fit, and accepted the procedure. Conversely, early progression, persistent unresectability, or clinical deterioration accumulated in the non-surgical pathway. A conventional surgery-versus-no-surgery Kaplan–Meier comparison would consequently be affected by confounding by indication and immortal-time bias. IC may still have value as a chemo-selection strategy in carefully selected patients [29], but the present study cannot establish that subsequent surgery caused improved survival. Addressing this question would require a larger prospective cohort or a design using time-dependent treatment modelling and adequate adjustment for baseline prognosis.

In the rapidly evolving landscape of neoadjuvant and perioperative immunotherapy—highlighted by pivotal trials such as KEYNOTE-689—the clinical role of conventional cytotoxic IC must be critically re-evaluated [24,25]. While perioperative pembrolizumab has demonstrated significant event-free survival improvements, conventional chemotherapy with TPF or TP carries substantial response heterogeneity and lacks a proven overall-survival advantage in resectable oral cavity cancer [7,8,9,11,25]. Nevertheless, cytotoxic IC may retain an individualised role in selected patients with rapidly progressive, symptomatic or bulky disease requiring tumour reduction before definitive local treatment, or in healthcare settings where perioperative immunotherapy is contraindicated or inaccessible [13,23]. The role of perioperative pembrolizumab in patients with PD-L1 CPS < 1 remains less clearly established because the current regulatory indication applies to CPS ≥ 1 disease [25,26].

This study has several limitations. Its retrospective, single-centre design and small cohort limited statistical power, particularly for regimen and survival analyses. Due to the 15-year study duration (2009–2024), treatment strategies inevitably evolved alongside advancements in diagnostic MRI, reconstructive surgical techniques, and modern radiotherapy planning. Treatment era was identified as a major potential confounding factor; for instance, PUL–bleomycin regimens were utilised predominantly during the earlier treatment era, whereas TPF and TP predominated in recent years. Although the small sample size precluded multivariable Cox regression adjusted for treatment era, this historical heterogeneity is explicitly emphasised as a study limitation that prevents direct causal comparison between regimens. We explicitly acknowledge that evaluating conventional induction chemotherapy (IC) regimens such as TPF and TP in OCSCC offers limited novelty regarding systemic drug development, as these regimens have been investigated for decades. However, the contemporary clinical value and primary novelty of this study lie in its granular, patient-level evaluation comparing the response of the largest baseline node against total nodal burden. Our finding that the single dominant node decreases significantly while the total nodal burden does not highlights critical limitations in relying on single-node short-axis measurements for post-induction response assessment in advanced OCSCC.

## 5. Conclusions

Curative-intent IC produced heterogeneous and generally modest unidimensional changes in primary advanced OCSCC. The largest baseline cervical node decreased significantly, whereas the total nodal burden did not, and neither nodal endpoint differed significantly from primary-tumour change. Radiological shrinkage was not associated with OS in exploratory landmark analyses, and no difference between TP and TPF was demonstrated. Survival findings and post-IC treatment pathways remain descriptive and do not establish a causal benefit from surgery. Prospective studies should combine standardised imaging with pathological response, toxicity, biomarkers, surgical outcomes, and contemporary perioperative systemic therapy.

## Figures and Tables

**Table 1:** Baseline and treatment characteristics.

Characteristic	Value
Patients	38
Age, median (range), years	56.5 (37.3–78.1)
Male sex	35 (92.1%)
Primary site	
Buccal mucosa	14 (36.8%)
Tongue	9 (23.7%)
Lower gingiva/alveolus	5 (13.2%)
Upper gingiva/alveolus	4 (10.5%)
Retromolar trigone	3 (7.9%)
Hard palate	2 (5.3%)
Upper Gum	1 (2.6%)
Clinical T stage	
T2	2 (5.3%)
T3	4 (10.5%)
T4a	15 (39.5%)
T4b	17 (44.7%)
Clinical N stage	
N0	11 (28.9%)
N1	2 (5.3%)
N2a	1 (2.6%)
N2b	9 (23.7%)
N2c	2 (5.3%)
N3b	13 (34.2%).
IC regimen: TP	20 (52.6%)
IC regimen: TPF	14 (36.8%)
IC regimen: PUL–Bleomycin	4 (10.5%)
Post-IC surgery	24 (63.2%)
Median IC cycles	3

**Table 2:** Paired radiological changes.

Endpoint	*n*	Baseline Median mm	Post-IC Median mm	Median Change %	Paired *p*
Primary tumour	25	55.0	46.0	−13.514	0.049
Largest baseline node	19	17.0	13.0	−16.667	0.004
Total nodal burden	19	28.0	30.0	−8.333	0.257

**Table 3:** Comparison of primary-tumour and nodal changes.

Comparison	*n*	Paired Difference *p*	Spearman Rho	Correlation *p*
Tumour vs. largest node	19	0.073	0.435	0.063
Tumour vs. total nodal burden	19	0.860	0.395	0.094

**Table 4:** Survival outcomes.

Outcome	Estimate
Deaths, all causes	16.0
Disease-related deaths	13.0
Other-cause deaths	3.0
Median follow-up, months	40.4
Median OS, months	45.1
1-year OS, %	85.3
3-year OS, %	63.1
5-year OS, %	35.6

## Data Availability

Data are not available for privacy reasons.
